# Blood donation trends in Zhejiang Province, 2017–2025: an ecological study before, during, and after COVID-19

**DOI:** 10.3389/fpubh.2026.1905236

**Published:** 2026-08-31

**Authors:** Guihua Jiang, Yani Ye, Hongyun Wang, Jinhui Wu, Jinhui Liu, Jun Xu

**Affiliations:** 1 Institute of Blood Transfusion, Jiaxing Central Blood Station, Jiaxing, Zhejiang, China; 2 Blood Center of Zhejiang Province, Hangzhou, Zhejiang, China

**Keywords:** blood donation characteristics, blood supply, COVID-19 pandemic, ecological study, trend analysis, Zhejiang Province, China

## Abstract

**Background:**

Voluntary blood donation is fundamental to ensuring a safe and adequate blood supply for clinical transfusion. The COVID-19 pandemic posed unprecedented challenges to global blood collection systems, including those in China. This ecological study aimed to characterize long-term blood donation patterns in Zhejiang Province from 2017 to 2025, spanning the pre-pandemic, pandemic, and post-pandemic periods.

**Methods:**

The blood donation data from 2017 to 2025 were obtained from the BIS 3.0 database maintained by the Blood Center of Zhejiang Province. These data included the number of donations, donated volume, donor demographics, as well as blood supply volume. A descriptive ecological design was employed for analysis.

**Results:**

The blood donation rate per 1,000 population, the total number of blood donations, and whole blood donation indicators exhibited an upward trend from 2020 to 2022, followed by a decline in the post-pandemic period. The male-to-female donor ratio (≈3:2) remained stable. The proportion of young donors (18≤ to <25 years) declined by 46.2%. Conversely, donors aged 55≤ to <60 years increased from 1 to 3%, representing the largest relative growth among all age groups. Among occupational groups, the proportions of employees, medical staff, and workers returned to earlier levels after the pandemic, whereas the proportion of students recovered more slowly. The supply of red blood cells and plasma declined after 2022, while platelet supply continued to increase.

**Conclusion:**

These results reveal marked shifts in blood donation dynamics temporally associated with the pandemic and its aftermath. Therefore, targeted incentives, public awareness, and trend monitoring are warranted to enhance the resilience of the blood supply system.

## 1 Introduction

Blood transfusion is essential for clinical treatment, emergency rescue, and surgical operations (1–3). As a finite and irreplaceable biological resource, the safety and adequacy of the blood supply are paramount for healthcare quality. With growing clinical demand, the current blood supply relies heavily on the voluntary, non-remunerated donation model (4). Since the promulgation of the *Blood Donation Law of the People’s Republic of China* in 1998, China has stipulated that healthy citizens aged 18–55 (extendable to 60 for eligible individuals) voluntarily donate blood (5, 6). In China, voluntary non-remunerated blood donation (VNRBD) is legally defined as comprising whole blood and apheresis platelets donations (7). As the cornerstone of clinical blood supply, VNRBD transcends its logistical role to serve as a vital indicator of social civility and public health resilience. Trend analysis was employed to characterize the direction, rate, and patterns (e.g., stability, growth, or volatility) of changes in annual blood donation data, offering insights into the descriptive characteristics of blood donation behavior dynamics (8). This approach provides a scientific basis for enhancing donor mobilization strategies and improving its adaptability. Furthermore, it helps elucidate both the short- and long-term impacts of public health crises on voluntary blood donation behavior.

Zhejiang Province, located in eastern China, is characterized by a developed economy and dense medical resources. Its blood donation patterns share common features with other developed coastal regions (9, 10). In December 2019, the first case of COVID-19 was reported in Wuhan, China. Thereafter, the WHO declared the outbreak a global pandemic in March 2020 (11). Starting in 2020, China rolled out phased lockdown and control measures. The State Council announced the lifting of Class A infectious disease control measures for COVID-19 in January 2023, marking the official transition to the post-pandemic phase (12). Accordingly, based on this administrative timeline of China’s pandemic response, we defined three phases: pre-pandemic (2017–2019), intra-pandemic (2020–2022), and post-pandemic recovery (2023–2025). We acknowledge that this administrative classification may not fully align with the epidemiological peaks of COVID-19 infections in China, which peaked in late 2022. The COVID-19 pandemic was a worldwide public health crisis that broadly shaped public health behaviors, socioeconomic activities, and the healthcare delivery system and placed considerable strain on blood collection and transfusion services (13, 14).

While many studies have focused on the immediate impact of COVID-19, few have tracked the full recovery process. To our knowledge, this is the first study to analyze blood donation dynamics across the entire pandemic cycle (2017–2025) in Zhejiang Province. Using continuous data spanning from the pre-pandemic baseline to the end of the recovery phase in 2025, we provide a comprehensive view of how the blood supply system adapted over the study period. Our findings offer practical insights to help develop timely and effective response strategies for future public health crises.

## 2 Materials and methods

### 2.1 Data collection and processing

Blood donation and supply data spanning 2017–2025 were obtained from the BIS 3.0 (Blood Information System 3.0) database. BIS 3.0 is operational administrative database developed by the Blood Center of Zhejiang Province. It functions as the central repository for blood service data across all 11 prefecture-level cities in Zhejiang Province, standardizing workflows ranging from donor recruitment and blood collection to laboratory testing, component preparation, and clinical issuance. The system complies with national data standards issued by the National Health Commission of China, ensuring data integrity and traceability. Given that BIS 3.0 is a production-level administrative database, data integrity was enforced at the point of entry. Critical fields, including donation ID, collection volume, and donation date, are mandatory system requirements; records with missing essential values are prohibited from being saved in the database. Furthermore, the system architecture utilizes unique donation identifiers and real-time validation logic (e.g., physiological range checks for blood volume and logical date sequencing) to prevent duplicate entries. Consequently, no missing data imputation, deduplication procedures, or outlier exclusion was required prior to analysis. The dataset was exported as a complete and validated aggregate, requiring only a final sanity check to confirm the completeness of the extraction scope.

The dataset extracted for this study contained records for whole blood and apheresis platelet donations. This included the number of donations and total volumes collected, as well as donor details such as gender, age, and occupation. We also examined the annual supply of blood components, including red blood cell concentrates, plasma derived from whole blood processing (hereafter referred to as plasma), and platelet concentrates, to assess blood availability. COVID-19 convalescent plasma (CCP) records were not included in the routine donation counts or supply analyzes.

According to the Blood Donation Law of the People’s Republic of China, individuals between the ages of 18 and 55 are permitted to donate blood. Those who have multiple donation experiences may continue to donate until the age of 60. Based on this regulation, eligible donors were divided into five age groups: 18≤ to <25, 25≤ to <35, 35≤ to <45, 45≤ to <55, and 55≤ to <60. Permanent resident population data for Zhejiang Province were sourced from the Zhejiang Provincial Bureau of Statistics^1^ (15). The calculation formula for the blood donation rate per 1,000 population is as follows: Blood donation rate per 1,000 population = (Annual number of voluntary blood donations/Permanent resident population of the year) × 1,000. Permanent resident population figures for Zhejiang Province (2017–2025) were obtained from official statistical bulletins. Figures for 2017–2019 are inter-censal estimates derived from annual sample surveys. The 2020 figure (64.68 million) is a year-end estimate anchored to the Seventh National Population Census count (64,567,588; reference: 00:00, 1 November 2020) (16). Figures for 2021–2025 are post-censal inter-censal estimates based on 5‰ or 1% sample surveys. The ~10.6% increase from 2019 to 2020 reflects primarily a census-based upward revision correcting for historically under-enumerated populations, rather than actual demographic growth within a single year.

Trend analysis was employed as a descriptive tool to illustrate the direction and rate of change in blood donation metrics over time. Simple slope calculations were used to quantify these changes between selected years, providing a descriptive overview of observed patterns rather than inferential statistical evidence.

### 2.2 Ethical approval

This study was approved by the Medical Ethics Committee of Jiaxing Central Blood Station (Approval No.: 2025-001), and informed consent was waived due to the retrospective analysis of anonymized administrative data.

## 3 Results

### 3.1 Trends in blood donation-related indicators from 2017 to 2025

**Figure 1:**
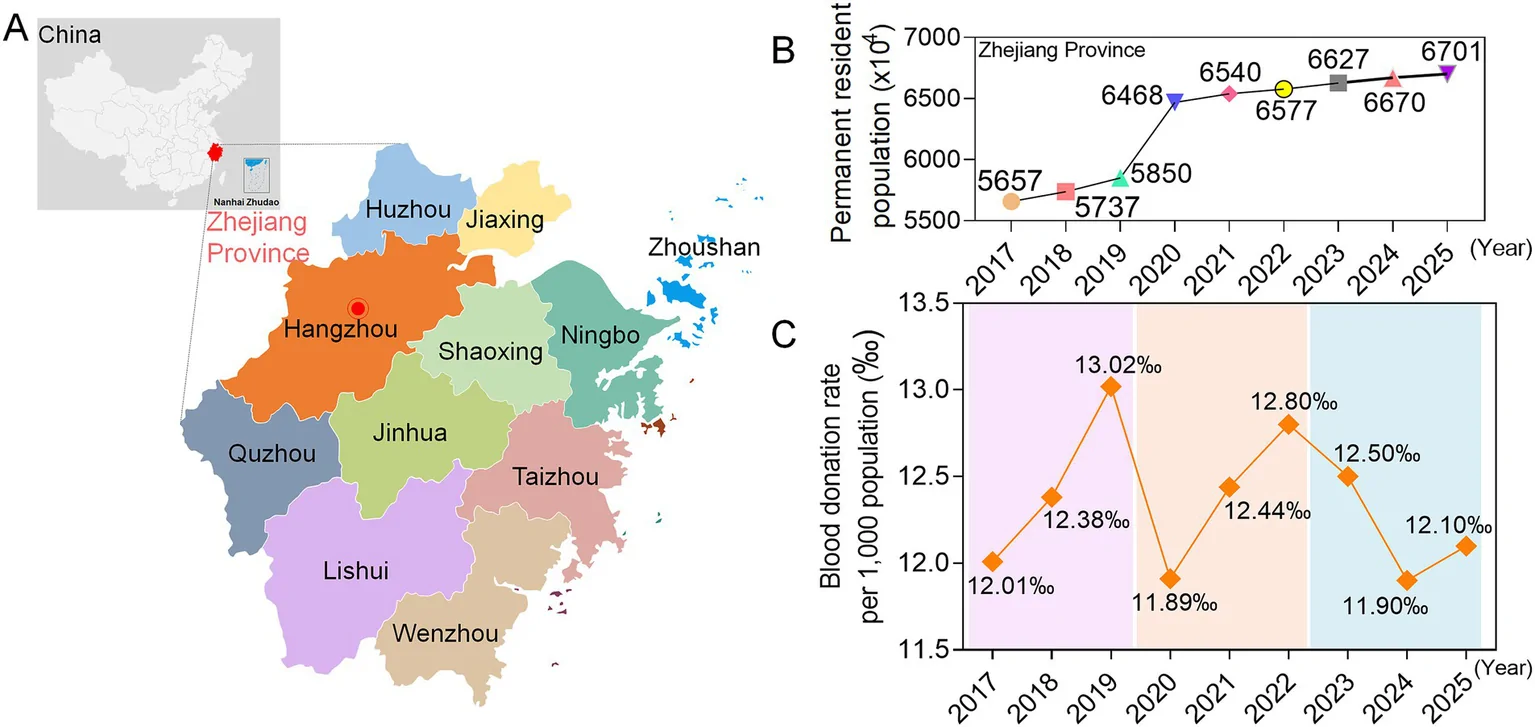
Trend in blood donation rate per 1,000 population **(A)** Map of Zhejiang Province and its 11 prefecture-level cities. **(B)** Permanent resident population of Zhejiang Province. Data sourced from the Zhejiang Provincial Bureau of Statistics. **(C)** Blood donation rate per 1,000 population in Zhejiang Province. Calculation formula: Blood donation rate per 1,000 population = (Annual number of voluntary blood donations/Permanent resident population of the year) × 1,000. Population denominators: 2017–2019, inter-censal estimates; 2020, year-end estimate benchmarked to the Seventh National Population Census (reference: 00:00, 1 November 2020); 2021–2025, post-censal estimates. The 2019 → 2020 discontinuity reflects a census-driven denominator revision rather than actual demographic growth.

Zhejiang Province is one of the most economically developed and open provinces in eastern China. It is composed of 11 prefecture-level cities, including Hangzhou, Ningbo, Wenzhou, Jiaxing, Huzhou, Shaoxing, Jinhua, Quzhou, Zhoushan, Taizhou, and Lishui (Figure 1A). Data released by the Zhejiang Provincial Bureau of Statistics showed that the number of permanent residents in the province grew continuously from 2017 to 2025 (Figure 1B). The blood donation rate per 1,000 population is an important indicator of clinical blood supply capacity. Generally, a higher rate corresponds to greater reflects a stronger capacity to ensure timely blood availability for medical needs. Statistics showed that the blood donation rate per 1,000 population fluctuated between 11.90‰ and 13.02‰ from 2017 to 2025. After peaking at 13.02‰ in 2019 and recovering to 12.80‰ by 2022, the rate entered a clear downtrend, falling markedly to 12.50‰ in 2023 and hitting a low of 11.90‰ in 2024 before a slight rebound to 12.10‰ in 2025 (Figure 1C).

**Figure 2:**
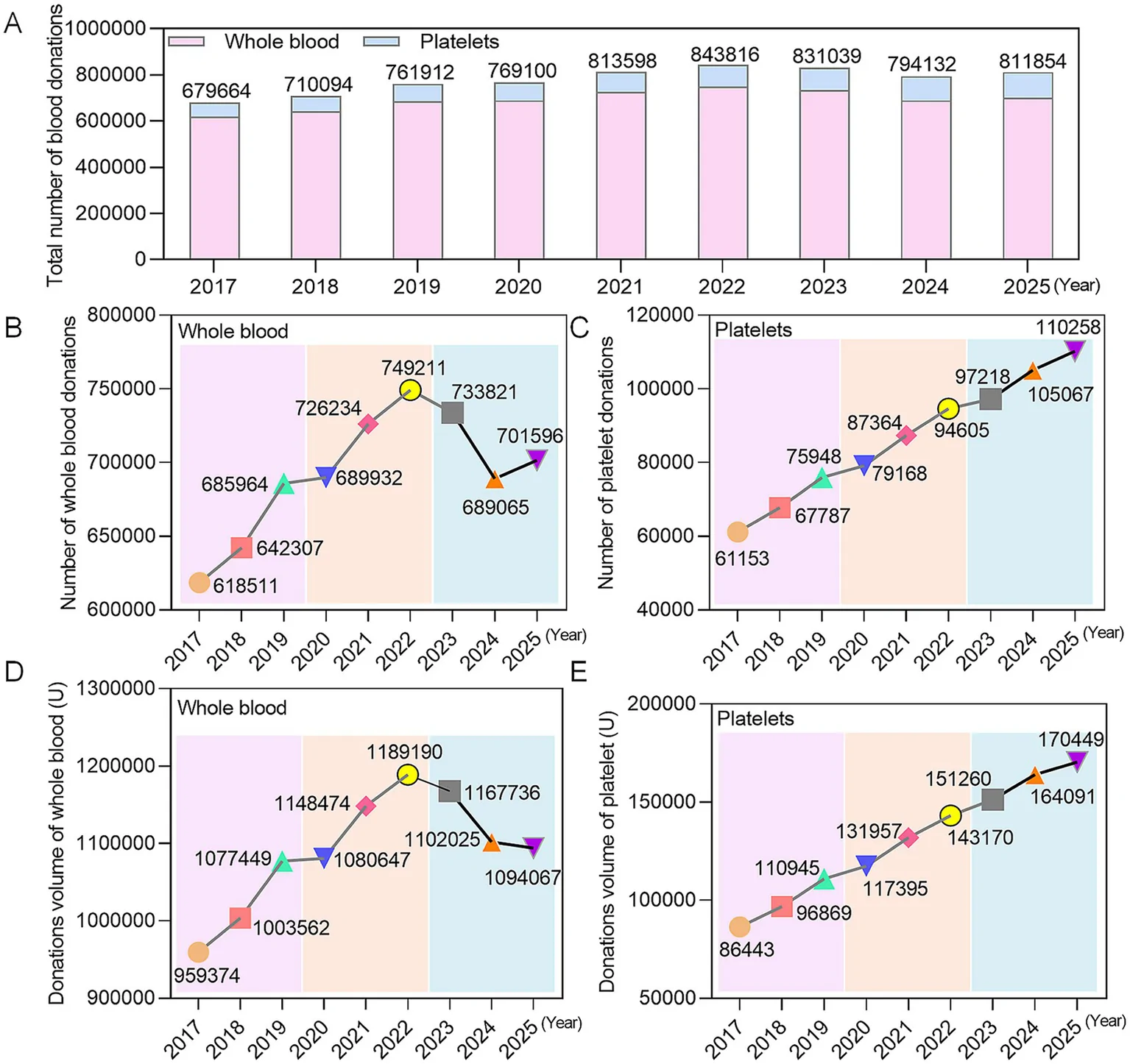
Number and volume of blood donations. **(A)** Total number of blood donations. **(B)** Number of whole blood donations. **(C)** Number of platelet donations. **(D)** Volume of whole blood donations. **(E)** Volume of platelets donations.

The overall trend for the total number of blood donations from 2017 to 2025 exhibited a pattern of initial growth, a mid-period decline, and a subsequent recovery (Figure 2A). This pattern was reflected in the trend of the number of whole blood donations (Figure 2B). In contrast, the number of platelet donations steadily increased from 2017 to 2025, representing a cumulative growth of approximately 80.3% (Figure 2C). Regarding donation volumes, whole blood collection volumes rose steadily from 2017 to 2022 before entering a phase of continuous decline through 2025, though the decline moderated in the final year (Figure 2D). Conversely, platelet donation volumes showed consistent accumulation from 2017 to 2025, achieving a net increase of approximately 97.2% (Figure 2E).

### 3.2 Blood donor characteristics from 2017 to 2025

**Figure 3:**
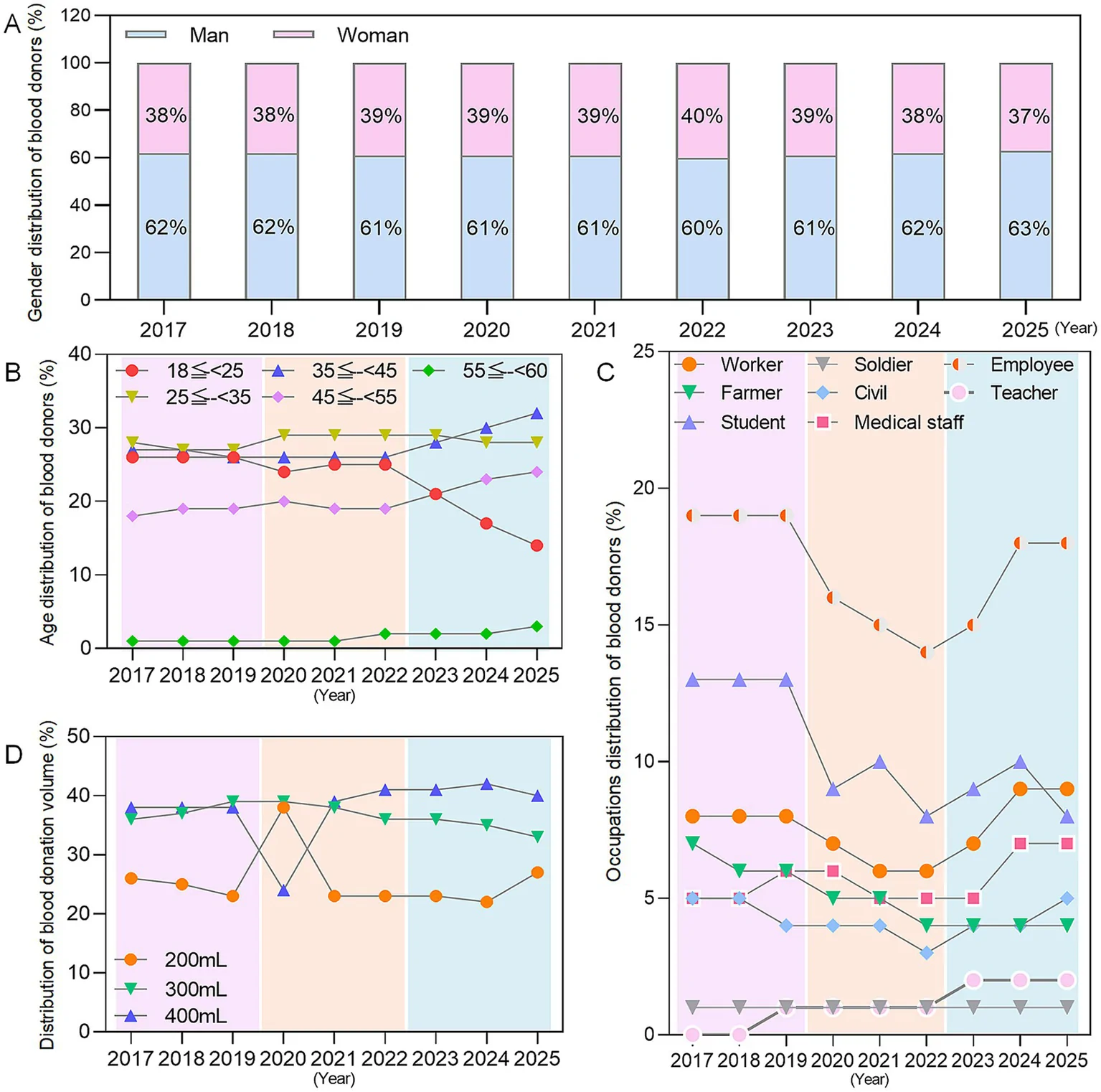
Characteristics analysis of blood donors from 2017 to 2025. **(A)** Gender distribution of donors. **(B)** Age distribution of donors. **(C)** Occupational distribution of donors. **(D)** Distribution of single donation volume.

From 2017 to 2025, the sex distribution of blood donors remained generally stable, with a consistent male-to-female ratio of 3:2 (Figure 3A). Donors were categorized into five age groups: 18≤ to <25, 25≤ to <35, 35≤ to <45, 45≤ to <55, and 55≤ to <60 (Figure 3B). The proportion of young donors (18≤ to <25 years) declined from 26% (2017) to 14% (2025), a 46.2% relative decrease. Conversely, middle-aged donors dominated the pool: the 35≤ to <45 group rose markedly from 27 to 32% (20.1% relative increase from 2017), while the 45≤ to <55 group increased steadily from 18 to 24% (31% relative increase from 2017). Donors aged 55≤ to <60 years expanded slowly but consistently from 1 to 3%, representing the largest relative gain among all age groups. Excluding unclassified occupations (Figure 3C), employees constituted the largest group of donors, with an average proportion of 17% during 2017–2025. Their share fell to a nadir of 14% during 2020–2022, then recovered sequentially to stabilize at 18% through 2023–2025. Workers and medical staff showed similar trends. Notably, the proportion of student donors stood at 13% pre-pandemic but fluctuated at low levels during the pandemic, bottoming out at 8% in 2022. Although it rebounded to 10% in 2024, the proportion fell back to 8% in 2025, remaining below the pre-pandemic baseline (Figure 3C).

From 2017 to 2025, the proportion of single-time 300 mL whole blood donations remained stable. In 2020, the proportions of 200 and 400 mL donations showed opposite trends. After the COVID-19 outbreak, 400 mL donations increased markedly, while 200 mL donations declined sharply. Subsequently, these divergent trends reversed gradually, with 400 mL donations trending downward and 200 mL donations edging upward through 2025 (Figure 3D).

### 3.3 Supply trends of blood components from 2017 to 2025

**Figure 4:**
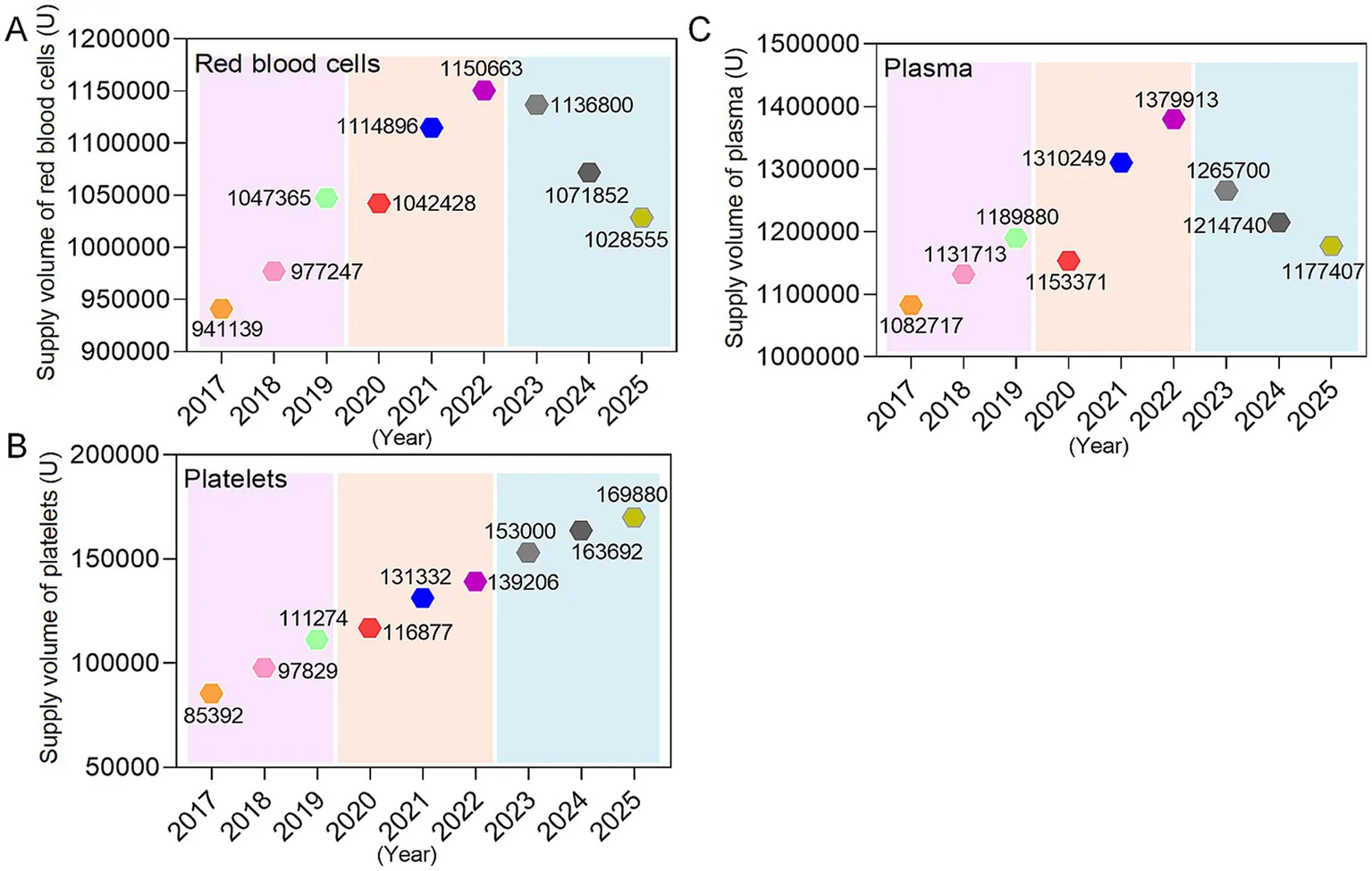
Characteristics of blood component supply. **(A)** Supply volume of red blood cell. **(B)** Supply volume of platelets. **(C)** Supply volume of plasma.

Red blood cell (RBC) supply rose from 941,139 units in 2017 to a peak of 1,150,663 units in 2022, then declined to 1,028,555 units by 2025 (Figure 4A). Notably, RBC supply dipped slightly to 1,042,428 units in 2020 (the initial COVID-19 outbreak period), approximately 0.5% below the 2019 level. In contrast, platelet supply exhibited continuous growth throughout 2017–2025, surging from 85,392 to 169,880 units (a nominal increase of approximately 99%, nearly doubling over the study period) (Figure 4B). Plasma supply paralleled that of red blood cells, peaking in 2022 before receding to 1,177,407 units in 2025 (Figure 4C). Crucially, these trends should be considered alongside changes in donation volumes and underlying clinical demand. The synchronous peaks in RBC and plasma supplies in 2022 coincided with the marked increase in overall blood donations (Figure 2).

## 4 Discussion

This study describes blood donation trends in Zhejiang Province from 2017 to 2025, covering the full trajectory of the COVID-19 pandemic. Significant challenges to blood donation systems during the COVID-19 pandemic have been reported in previous studies (17). For example, during the early stages of the outbreak, blood donations in Tokyo declined drastically (18). The World Health Organization (WHO) also noted a 20–30% decrease in blood supplies worldwide (19). Our research found that several key blood donation indicators in Zhejiang Province increased annually during the COVID-19 pandemic. These indicators included the number of donations, total blood donation volume, and the volume of supplied blood components. However, these indicators (apart from platelet-related metrics) exhibited a declining trend in the 2 years after the end of the pandemic. This pattern potentially suggests a temporal association between the broader COVID-19 era and voluntary blood donation in Zhejiang Province. We hypothesize that this delayed effect may relate to both the government’s policy response and potential psychological changes among the public.

Critically, China experienced its most substantial infection wave immediately following the optimization of zero-COVID policies in December 2022–January 2023. This wave occurred at the very onset of the period we designated as “post-pandemic recovery” (2023–2025). Consequently, the observed decline in donation indicators during 2023 may represent either: (a) a direct acute impact of this massive infection wave on donor availability and willingness, (b) a manifestation of the “delayed effect” of the pandemic as we proposed, or (c) a combination of both. The annual aggregated nature of our data and the ecological study design prevent us from disentangling these competing explanations at the individual donor level. However, the temporal coincidence of the 2023 dip with this infection wave suggests that acute infection-related disruptions likely played a substantial role, potentially mask or confounding any longer-term “delayed behavioral effect.” We therefore frame the post-2022 trends cautiously as temporally associated with the broader pandemic era, rather than attributing them definitively to a singular causal mechanism. At the beginning of the pandemic, the Zhejiang government took rapid measures to stabilize the blood supply. These measures included expanding media publicity, extending donation center service hours, and introducing strategies like online appointments and staggered time slots for donors (9). After COVID-19 prevention and control policies were optimized, middle-aged donors showed renewed willingness to donate blood. These combined efforts helped ensure a stable blood supply. Similar blood donation trends were observed in countries such as Iran and Japan (20, 21).

Notably, the 2025 rebound in both the per-1,000-population donation rate and whole blood donation counts may be partially attributable to voluntary blood donation incentive measures successively rolled out across Zhejiang Province since 2023 (22, 23). Representative examples include *Quzhou’s Several Policy Measures to Encourage Voluntary Blood Donation* (Quzhengbanfa [2023] No. 28), which grants biennial free health examinations to “Camphor Award” recipients (22), and *Jiaxing’s Several Preferential Measures to Encourage Voluntary Blood Donation* (formalized in June 2024), which comprises a 12-item benefit package covering free cinema admission, fitness discounts, and housing provident fund loan preferences (23). While these initiatives likely bolstered donor motivation, the ecological nature of this study precludes direct quantification of their individual contributions.

Globally, women account for approximately 33% of blood donors, with proportions falling below 10% in some countries (24). The male-to-female ratio of Zhejiang Province’s permanent population stayed constant between 2017 and 2025, ranging from 1.05:1 to 1.09:1. However, the proportion of male to female blood donors remained constant at 3:2 during that time. This observation implies that women in Zhejiang were persistently less likely than men to donate blood. Possible contributing factors, which were not directly measured in this study, might include worries about menstrual anemia, needle fears, anxiety about potential negative reactions, and a lack of knowledge about the significance and safety of blood donation (25). Targeted measures are required to increase female participation. These could include educational initiatives that focus on physiological issues that affect women, including anemia brought on by menstruation. Other strategies can include providing post-donation iron supplements to allay worries about iron deficiency and lowering anxiety through open communication.

Between 2017 and 2025, the age composition of blood donors in Zhejiang Province underwent a profound structural transformation, as evidenced by absolute donor counts and relative age proportions (Supplementary Table S1 and Figure 2B). The youngest cohort (18≤ to <25) experienced a sharp decline of 37.9% (from 178,029 in 2017 to 110,501 in 2025), with the most precipitous reduction occurring after 2022. This decline corresponds to a 13% reduction in the proportion of this group, suggesting a persistent and generalized erosion of youth participation across the donor base. This trend may be partially explained by contextual factors prevalent during the pandemic. Firstly, with a high proportion of students in this age group, it is plausible that heightened health concerns among families (e.g., following infections or vaccinations) potentially led to increased parental hesitation regarding adolescent blood donation. Secondly, the observed decline may reflect a reliance on organized recruitment (e.g., school campaigns) rather than intrinsic motivation for regular donation among youths (19). In contrast, older age groups showed marked expansion: the 35≤ to <45 group increased by 44.2% (181,944–262,407), surpassing all other groups to become the largest donor segment by 2025. The 45≤ to <55 and 55≤ to <60 group grew by 56.5 and 350.9%, respectively. This divergence resulted in a complete reordering of the donor age distribution: the 18≤ to <25 group fell from the largest cohort in 2017 to the fourth-largest in 2025. These absolute-number trends provide robust evidence of a genuine and pronounced aging of the donor pool, driven primarily by the accelerating decline in youth participation and the sustained increase in middle-aged and older donor involvement. Notably, the highly stable gender ratio observed throughout 2017–2025 (Figure 2A; consistently ~61% male vs. 39% female) suggests this downward trend among youth is a generalized phenomenon affecting the broader donor population, rather than a sex-specific pattern.

In response to this situation, age-specific recruitment strategies should be developed. For young people, schools can include blood donation education in courses and campus activities. Sharing real-life stories about donation outcomes may help inspire participation. For all age groups, donation centers should improve the overall experience. Measures such as simplifying the donation process, creating comfortable environments, and offering personalized appreciation can help encourage repeat donations.

Voluntary blood donation is very important to public health. The COVID-19 pandemic was associated with notable changes in blood donation patterns across Zhejiang Province. This study documents these shifts and highlights key features of the blood supply system under stress, offering insights into how such systems may respond differently during large-scale public health disruptions.

## 5 Study limitations

This ecological study is subject to several inherent limitations that constrain causal inference. First, reliance on annually aggregated provincial data from the BIS 3.0 system precluded individual-level behavioral analysis and longitudinal tracking (e.g., distinguishing first-time from repeat donors). Consequently, the observed trends serve as descriptive insight rather than inferential statistical evidence. Second, critical methodological discontinuities limit specific temporal comparisons. The 2019–2020 boundary is particularly problematic: the 2020 population denominator reflects a census-driven revision, whereas 2017–2019 figures are based on inter-censal surveys. This statistical artifact complicates the interpretation of the 2019–2020 donation rate change, necessitating cautious interpretation and suggesting that trend analyzes are most reliable when conducted within distinct epochs (e.g., 2017–2019 vs. 2021–2025). Furthermore, the inability to disentangle the acute impact of the December 2022–January 2023 infection wave from potential longer-term behavioral effects of the pandemic represents a major source of temporal confounding, limiting our ability to attribute post-2022 trends to a singular causal mechanism. Third, generalizability is constrained geographically; while findings are representative of developed coastal regions like Zhejiang, extrapolation to inland provinces with differing demographic and healthcare profiles requires caution.

Future studies should address these gaps by incorporating multi-provincial datasets. Specifically, individual-level linkage studies tracking donor infection status and donation behavior are warranted to disentangle the overlapping influences of acute infection waves and long-term pandemic effects. Additionally, implementing sex-stratified and age-disaggregated demographic analyzes will facilitate the development of targeted recruitment strategies tailored to specific population subgroups.

## 6 Conclusion

In summary, this observational study describes the dynamics of Zhejiang’s blood donation system from 2017 to 2025. We observed temporal alignments between pandemic phases and fluctuations in whole blood metrics, alongside shifts in donor age and occupation profiles. Given the ecological design, these findings preclude definitive causal inferences but underscore the necessity of targeted recruitment and routine monitoring to enhance system resilience.

## Data Availability

The raw data supporting the conclusions of this article will be made available by the authors, without undue reservation.

## Footnotes

1 https://tjj.zj.gov.cn/.
