# Supplementary material for: Blood donation trends in Zhejiang Province, 2017–2025: an ecological study before, during, and after COVID-19
Source: Front Public Health. 2026 Aug 31;14:1905236. doi: 10.3389/fpubh.2026.1905236 (PMC13572587; doi:10.3389/fpubh.2026.1905236)
Supplement: Supplementary file 1 [file Data_Sheet_1.PDF]

Supplementary Table S1 Table 1 Number of blood donors by age group, Zhejiang Province, 2017–2025

Age group (years)	18≦-<25	25≦-<35	35≦-<45	45≦-<55	55≦-<60
2017	178029	190689	181944	123962	4542
2018	186794	194204	188691	134804	5409
2019	199083	207958	199249	147676	7946
2020	184423	221054	202605	151627	9391
2021	203533	232880	210693	155357	11130
2022	207897	242051	220857	160074	12937
2023	174314	237876	234153	170408	14288
2024	135876	220333	239213	180169	16647
2025	110501	224393	262407	194078	20475
